# Epidemiological, pathological and microbiological investigation of neonatal lamb mortality in the region of Constantine, Algeria

**DOI:** 10.1002/vro2.70021

**Published:** 2025-09-13

**Authors:** Hayem Benmebarek, Samia Djeffal, Louiza Benhamza, Omar Bouaziz, Sarra Medjedoub, Mustapha Adnane Smadi, Houda Boufendi, Asma Temime, Jean‐Marc Rolain, Seydina M. Diene

**Affiliations:** ^1^ GSPA Research Laboratory (Management of Animal Health and Productions), Institute of Veterinary Sciences University Frères Mentouri Constantine Algeria; ^2^ Department of Agronomy University 20 Août Skikda Algeria; ^3^ Biotechnology Research Center (CRBT) Constantine Algeria; ^4^ APHM, MEPHI, IHU‐Méditerranée Infection, Faculté de Pharmacie Aix Marseille University Marseille France

**Keywords:** affected organs, lamb, lesions, neonatal mortality, pathogens, risk factors

## Abstract

**Background:**

Neonatal lamb mortality affects the sheep flock's productivity. In Algeria, data are very limited, making investigations quite relevant. This study aimed to determine risk factors associated with neonatal lamb mortality, describe gross and histopathological lesions on affected organs, and identify bacterial pathogens involved.

**Design:**

Thirty‐five sheep farms located in Constantine participated in this survey between February and December 2022. An epidemiological questionnaire was used to describe monitoring practices and to identify associated risk factors. Twenty‐two collected lamb corpses (immediate postmortem changes) underwent detailed postmortem examinations, including anatomopathological, histopathological and microbiological analyses.

**Results:**

The recorded neonatal mortality rate was 5.74%. Statistical analysis revealed significant associations between mortality and factors such as soil and livestock building hygiene, as well as the breeding system used (*p *< 0.05). The lungs, liver, kidneys and heart were the most affected organs. Causes of death included respiratory disorders, omphalitis with septicaemia, stillbirth, trauma and starvation‐hypothermia syndrome. From these organs, 218 bacterial strains were isolated, mainly Enterobacteriaceae (*Escherichia coli, Klebsiella pneumoniae, Providencia* spp., *Citrobacter* spp.), causing primary infections, alongside non‐fermenting Gram‐negative bacilli (*Pseudomonas aeruginosa, Comamonas* spp., *Acinetobacter* spp.), responsible for the associated septicaemic evolution. According to the EUCAST breakpoints, isolates were resistant to commonly used antibiotics: amoxicillin, amoxicillin/clavulanic acid, trimethoprim/sulfamethoxazole and tetracycline. Notably, only *Klebsiella* spp. strains harboured *bla*_SHV_ genes.

**Conclusion:**

This study concluded that improved sanitary protocols, early diagnosis and antimicrobial testing are crucial to prevent neonatal mortality. It also recommended larger studies and farmer training programmes to enhance lambing and neonatal care practices.

## INTRODUCTION

The production of red meat in Algeria is largely based on sheep farming, which accounts for a significant portion of the country's agricultural output.^1^, ^2^ The profitability of livestock farming can be affected by neonatal mortality, leading to considerable economic losses.^3^ According to reports from the UK, economic losses are around 20–25£ per lamb when mortality occurs during gestation.^4^ Due to their impact on the annual yield, many studies have been conducted on neonatal mortality to determine their rate, associated risk factors, causes (infectious or non‐infectious), and the organs most affected in different countries, including India,^5^ France,^6^ Morocco^7^ and several regions of Algeria, including Bordj Bou Arreridj, the Sahara and Tiaret.^8^, ^9^, ^10^, ^11^

In Algeria, most studies have shown that lamb mortality rates during the perinatal period vary from 13% to 25%.^8^, ^9^, ^10^, ^11^ Moreover, Gani and Niar found that the age of ewes was the risk factor implicated in mortality recorded in the Sahara,^9^ while in Bordj Bou Arreridj, lesion assessment revealed that the most affected systems were the respiratory, digestive and cardiac systems.^10^

Mortality occurring in the perinatal period is classified into three types: prenatal mortality, stillbirth occurring during lambing and postnatal mortality, categorised as immediate (from birth to 3 days), intermediate (from 3 to 7 days), and delayed (7 days until weaning),^12^ and they can be of an infectious or non‐infectious origin. Their occurrence is strongly associated with risk factors that can be ewe‐related (body condition, lambing season, age, maternal behaviour and colostrum quality),^12^, ^13^ lamb‐related (age, sex and weight),^12^ or environmental/management‐related (soil hygiene, livestock buildings, lambing in the sheepfold or outdoors, and breeding system).^6^, ^12^, ^13^

Infectious deaths may be due to different pathogens, such as bacteria. Microorganisms can generate several symptoms and lesions in the lamb's organs. Numerous Gram‐negative bacteria from the Enterobacteriaceae family were involved in lamb mortality. Some are frequently described, such as *Escherichia coli*, *Pasteurella* spp. and *Salmonella* spp.^8^, ^12^ Others are rarely reported, like the genus *Providencia*, which includes species that are either responsible for nosocomial infections, such as *P. stuartii*, or widely distributed in the environment, such as *P. entomophila*.^14^, ^15^, ^16^, ^17^

Although there have been some studies on lamb mortality in Algeria, data on neonatal lamb mortality are limited, which makes investigations on this issue quite relevant. This study aims to describe neonatal lamb mortality on farms located in the region of Constantine by identifying the risk factors associated with this mortality, determining the specific pathogens responsible, and providing a detailed description of the macroscopic and microscopic lesions observed in the viscera of affected lambs.

## MATERIALS AND METHODS

### Ethical approval

This study was approved by the El Khroub Scientific Council of the Institute of Veterinary Sciences of Constantine 1 Frères Mentouri University. Authorisations for the collection of lamb carcases (natural deaths) and the transfer of samples (bacterial strains and histological slides) to the Institut Hospitalo‐Universitaire (IHU) of Marseille, France, were issued under reference numbers 145/22 and 1072/22, respectively.

### Study area

The Constantine region is located at 36°21′54′′ north, 6°36′52′′ east in northeastern Algeria. Its area is 2187 km^2^, and it has an altitude of 574 m above sea level, including three geographical zones: the mountainous zone, the interior basins and the high plains. It is delimited by Skikda to the north, Guelma to the east, Oum El Bouaghi to the south, and Mila to the west. Constantine has a continental climate characterised by summer temperatures ranging from 25°C to 40°C, winter temperatures from 0°C to 12°C, and an annual precipitation of 400–600 mm per year.

### Sample collection

This longitudinal study took place between February and December 2022. The survey included 35 sheep farms located in the province of Constantine in the northeast of Algeria. We carried out samplings in a rigorous and organised manner. These farms were selected for their easy access, the possibility of collecting lamb carcases without delay, and the cooperation of their owners. The target population consisted of lambs ranging in age from a few hours to 60 days. A total of 33 lamb carcases were recovered from 10 of the 35 farms. However, only 22 of these carcases were found to be in good preserved condition and were therefore included in this study. The number of sheep farms and carcases is representative of breeding system characteristics of the study region, as per the authors and was limited by the capacity of the laboratory that processed the samples. The carcases were transported within a few hours of death (not exceeding 2 hours) on ice packs to the veterinary institute, where the postmortem examinations were performed.

## EPIDEMIOLOGICAL STUDY

### Data collection

The data were obtained from personal observations, owners’ data, and veterinarians who medically monitor the livestock by performing weekly visits to the farms during lambing seasons.

### Questionnaire

The epidemiological questionnaire addressed to the farms contained 76 closed‐type questions divided into two sections. The first section considered information about the rearing system, infrastructures, food, hygiene, care, supervision of the lambs in each farm, vaccination programme, and the use of antibiotics. The second section concerned reproduction, the number of lambing seasons, their periods, and the monitoring practices provided during lambing seasons.

### Statistical analysis

The collected survey database was used to calculate the mortality rate on farms and reported in contingency tables to determine potential risk factors. The potential risk factors that were selected according to the questionnaire were the hygiene of the soil and the livestock buildings. Reproductive management was considered through the reproduction system and the use of housing for lambs and ewes. Nutritional factors included the amount of feed available for the ewes. Management of the lambs was assessed through practices such as cleaning of the umbilical cord. Individual characteristics comprised age, sex, month and season of birth, and body condition score. These factors were analysed to estimate their association with neonatal lamb mortality records and the type of mortality (infectious or non‐infectious). Data were assessed by Fisher's exact test (95% confidence interval, *p *< 0.05) using the RStudio (version 7d165dcf, build 353, 3 December 2022).

## ANATOMOPATHOLOGICAL AND HISTOPATHOLOGICAL STUDY

### Postmortem examination

An initial inspection was required to evaluate the preservation condition of each lamb carcase. Any significant postmortem change led to the exclusion of the carcase from the study. Each selected carcase (showing only immediate postmortem changes) was linked to a report including information on the age from the farm register, sex, condition of preservation, nature of death, breeding management, body condition score, presence of nasal or ocular discharge, and the appearance of the mucous membranes. A postmortem examination was performed on the 22 selected lamb carcases, involving a visual inspection of the organs, palpation and systematic incisions of the organs to determine the cause of death (infectious or non‐infectious).^18^, ^19^, ^20^, ^21^ Sixteen of the 22 carcases showed severe lesions, suggesting an infectious cause of death. Samples (*n* = 155) from organs presenting lesions (lungs, liver, kidney, heart, spleen, muscle and intestines) and swabs were retained for histopathological (*n* = 65/155) and microbiological study (*n* = 90/155) (Table 1), and to perform antimicrobial susceptibility testing and detect resistance genes with PCR.

**TABLE 1:** Sample collection from the affected organs of the lamb carcases.

Lambs’ carcases	Sample collection from the affected organs	
	For histopathological study	For microbiological study
1	Intestines	Intestines
2	Liver	Liver
3	Lungs, liver, kidney, muscle of the hindlimb	Lungs, liver, kidney, muscle of the hindlimb
4	Lungs, kidney, abomasum	Lungs, kidney, abomasum
5	Lungs, liver, heart, kidney, muscle of the forelimb, muscle of the hindlimb	Lungs, liver, heart, kidney, muscle of the forelimb, muscle of the hindlimb
6	Lungs, liver, heart (pericardium and myocardium), kidney	Lungs, liver, heart (pericardium and myocardium), kidney, trachea (2 sections), tracheal swab (03)
7	Lungs, liver, heart, kidney	Lungs, liver, heart, kidney, trachea (2 sections), peritoneum swab
8	Lungs, liver, heart, kidney	Lungs, liver, heart, kidney, trachea (2 sections), tracheal swab (04)
9	Lungs, liver, heart, kidney, spleen (2 sections)	Lungs, liver, heart, kidney, spleen (2 sections), trachea, tracheal swab (03)
10	Lungs, liver, heart, kidney, spleen (2 sections)	Lungs, liver, heart, kidney, spleen (2 sections)
11	Lungs, liver, heart, kidney	Lungs, liver, heart, kidney
12	Lungs, liver, heart, kidney	Lungs, liver, heart, kidney
13	Lungs, liver, heart, kidney	Lungs, liver, heart, kidney
14	Lungs, liver, heart, kidney	Lungs, liver, heart, kidney
15	Lungs (2 sections), liver, heart, kidney	Lungs (2 sections), liver, heart, kidney
16	Lungs, liver, heart, kidney	Lungs, liver, heart, kidney, trachea (2 sections), tracheal swab (04), peritoneum swab
Total	65	90
Total samples	155	

### Slide preparation for histopathological study

After fixing the 65 organ samples in formaldehyde (MZK BactChim), haematoxylin and eosin (Sigma‐Aldrich) staining was performed, as described in the literature,^22^ to obtain slides observable under the microscope.

## Microbiological study

### Bacterial isolation and identification

Asepsis was practised as much as possible in collecting and handling the affected organ sections; the surface of the affected organs was sterilised before culture using a soldering spatula, and sections were placed in sterile containers and transferred to the laboratory in a cooler box without further delay.^23^

Bacterial strains were isolated and identified by culturing samples from the affected organs in a tryptic soy agar (Becton Dickinson), MacConkey agar (Becton Dickinson), and Columbia agar with 5% sheep blood (Becton Dickinson) for 24 hours at 37°C.^24^, ^25^ Suspected colonies were first identified using the API 20E System (bioMérieux), followed by MALDI‐TOF (Bruker Daltonics).^26^

### Antimicrobial susceptibility testing

The antibiotic susceptibility test was determined using Mueller–Hinton agar and the standard disk diffusion procedure described by the European Committee on Antimicrobial Susceptibility Testing (EUCAST, 2022).^27^ Sixteen antibiotics were tested for two families: the Enterobacteriaceae and the non‐fermenting Gram‐negative bacilli (Table 2).

**TABLE 2:** Antibiotics tested for the Enterobacteriaceae and the non‐fermenting Gram‐negative bacilli isolates.

Antibiotics tested for Enterobacteriaceae	Antibiotics tested for non‐fermenting Gram‐negative bacilli
Amoxicillin AMX (20 µg)	Ticarcillin TIC (75 µg)
Amoxicillin/Clavulanic acid AMC (20/10 µg)	Ticarcillin/Clavulanic acid TIM (85 µg)
Amikacin AK (30 µg)	Piperacillin/Tazobactam TZP (36 µg)
Piperacillin/tazobactam TPZ (36 µg)	Aztreonam ATM (30 µg)
Ceftriaxone CRO (30 µg)	Ceftazidime CAZ (10 µg)
Cefepime FEP (30 µg)	Cefepime FEP (30 µg)
Mecillinam MEC (10 µg)	Meropenem MER (10 µg)
Trimethoprim/Sulfamethoxazole SXT (25 µg)	Imipenem IPM (10 µg)
Fosfomycin FF (200 µg)	Fosfomycin FF (200 µg)
Furane FT (100 µg)	Rifampicin RA (5 µg)
Imipenem IPM (10 µg)	Trimethoprim/Sulfamethoxazole SXT (25 µg)
Ertapenem ETP (10 µg)	Amikacin AK (30 µg)
Gentamicin GN (10 µg)	Ciprofloxacin CIP (5 µg)
Ciprofloxacin CIP (5 µg)	Minocycline MIN (30 µg)
Tetracycline TET (30 µg)	Colistin CS (50 µg)
Colistin CS (50 µg)	Gentamicin GN (10 µg)

### PCR detection of resistance genes

All isolates were screened for resistance genes. For the extraction of total nucleic acids, we used the dry heat method as follows: colonies were put in a test tube containing 1 mL of distilled water and boiled for 10 minutes in a water bath.^28^

Because β‐lactams constitute a major group of antibiotics commonly used to treat severe infections, we limited our study to the detection of extended‐spectrum beta‐lactamase (ESBL) genes. Real‐time PCR was conducted using specific primers for ESBL genes, namely, *bla*_CTX‐M‐1_ group (CTX‐A),^29^ *bla*_CTX‐M‐9_ group (CTX‐B),^30^ *bla*_TEM_ group and *bla*_SHV_.^31^ The amplification protocol was performed as follows: denaturation at 50°C for 2 minutes, hybridisation at 95°C for 15 minutes, probe hybridisation at 95°C for 1 second, elongation at 60°C for 30 seconds, plate read, then step 35×, and finally cooling at 45°C for 30 seconds using the CFX96 Touch Real‐Time PCR Detection System (Bio‐Rad). The results were read using CFX Maestro software.

## RESULTS

### Epidemiological study

The livestock on the 35 farms was of the Ouled Djellal breed, and a semi‐extensive to extensive farming system was employed. Seven farms belonged to the public sector, while 28 belonged to the private sector; their rearing capacity varied from 50 to 886 sheep per farm. Due to the continuous rearing of rams with ewes, the majority of these farms (*n* = 27/35) had one or two mating seasons, while the remaining farms (*n* = 8/35) had two mating seasons per year. To prepare the animals for the breeding season, some farms performed flushing (feeding of extra concentrate to ewes 3 or 4 weeks before breeding) and crutching (removal of wool from the perineal area and the base of the tail of ewes). The methods used were flock mating and pen mating. During lambing seasons, the questionnaire revealed the use of traditional breeding techniques in the majority of farms and a lack of monitoring practices, such as assisting ewes during lambing and lambs during their first hours of life by ensuring that they stand and suck their mother's udder. Furthermore, veterinarians use broad‐spectrum activity antibiotics such as amoxicillin, amoxicillin/clavulanic acid, trimethoprim/sulfamethoxazole and tetracycline. Only two of the studied farms solicited a veterinarian in two cases of neonatal infections, and lambs were treated with tetracycline.

The ranking of these lamb deaths by the period they occurred in was as follows: stillbirth 0.69% (*n* = 4/574), immediate post‐natal period (first days after birth) 0.87% (*n* = 5/574), intermediate post‐natal period (1 week after birth) 1.57% (*n* = 9/574), and delayed post‐natal period (from a week after birth until weaning) 2.61% (*n* = 15/574). It shows that the delayed postnatal period had the highest mortality rate, and the most affected age category was 20 days old at 40% (*n* = 6/15).

During the study period, in a total population of 574 lambs at risk, the mortality rate was 5.74% (*n* = 33/574). These deaths were associated with several risk factors. Statistical analysis indicates that the hygiene of the soil and livestock buildings (*p *≤ 0.05) was the determining risk factor in recording lamb neonatal mortality. The evolution of this mortality (infectious or non‐infectious) was related to the breeding system (reproduction system [*p* = 0.002], housing ewes according to their stage of gestation [empty/full] [*p* = 0.02], housing lambs according to their age [*p* = 0.02]), and the hygiene of the soil and the livestock buildings (*p* = 0.01) (Table 3).

**TABLE 3:** Risk factors determining lamb mortality record and type of mortality (infectious/non‐infectious).

			Fisher's exact test	
Risk factors	Categories	Mortality rate (%)	Determining mortality record *p‐*value	Determining the type of mortality (infectious/non‐infectious) *p‐*value
Reproductive system	**2 Seasons per year**	**4.35**	0.07661	**0.002806**
	1 Season per year	1.39		
Housing ewes according to stage (empty/full)	Applied	1.39	0.3926	**0.02025**
	**Not applied**	**4.35**		
Housing lambs according to age	Applied	1.39	0.6614	**0.02025**
	**Not applied**	**4.35**		
Management of the lambs (cleaning of the umbilical cord)	Applied	1.39	0.2857	0.06155
	**Not applied**	**4.35**		
Hygiene of the soil and the livestock buildings	Excellent hygiene practices	1.04	**0.005591**	**0.01585**
	**Bad hygiene practices**	**4.70**		
Amount of feed available for the ewes	Sufficient	1.04	0.3811	1
	**Insufficient**	**4.70**		
Age	Stillbirth	0.69	/	2.528e‐05
	Immediate post‐natal period	0.87		
	Intermediate post‐natal period	1.57		
	**Delayed post‐natal period**	**2.61**		
Sex	**Male**	**3.48**	/	0.1824
	Female	2.26		
Month of birth	February	0.69	/	0.1356
	March	0.35		
	August	0.35		
	September	1.4		
	**October**	**2.61**		
	November	0.17		
	December	0.17		
Season of birth	Winter	0.87	/	0.6866
	Spring	0.35		
	Summer	0.35		
	**Autumn**	**4.18**		
Body score condition	Thin	1.57	/	1
	Average	0.52		
	**Fleshy**	**3.65**		

### Anatomopathological and histopathological study

**FIGURE 1:**
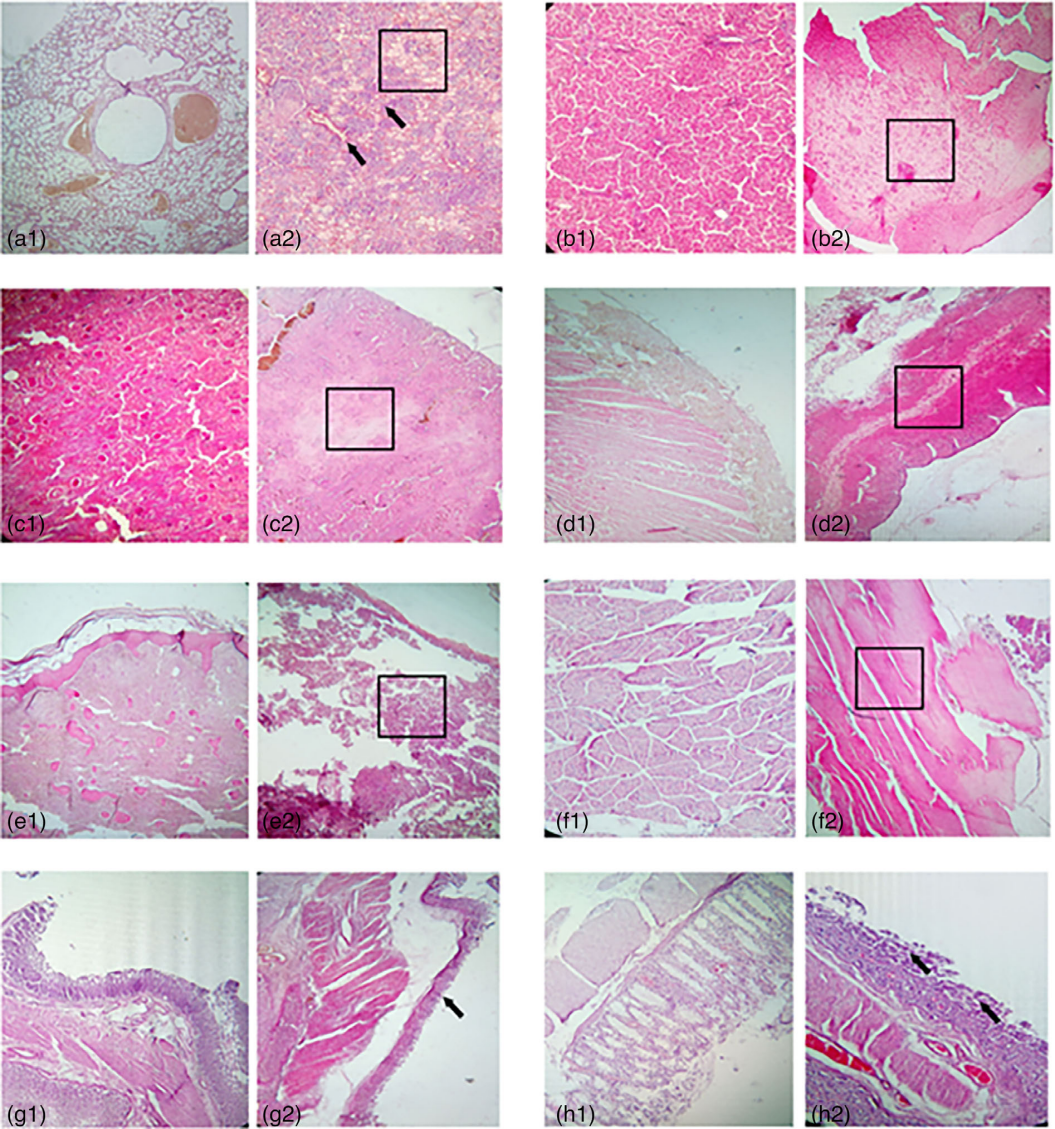
Observed lesions, lambs (×40). (a1–h1) Histological structure of the organs (lungs, liver, kidney, heart, spleen, muscle, abomasum and intestines). (a2) Congestion of the lung parenchyma with exudate (circle) and inflammatory foci (arrows). (b2, c2, d2) Diffuse necrosis on the liver and kidney parenchyma (square), and on the myocardium (circle). (e2) Destruction of the spleen parenchyma (square). (f2) Muscle necrosis (square). (g2, h2) Destruction of the abomasum and intestinal epithelium (arrows).

The first inspection to assess the preservation status of each lamb carcase excluded 11 out of 33 carcases due to severe postmortem changes. Postmortem examinations were conducted on 22 lamb carcases that were between a few hours and 30 days old. The lesion reports concluded that in six of the 22 carcases, the cause of death was non‐infectious. This included four cases of amniotic fluid aspiration, where amniotic fluid was found in the respiratory and digestive tracts, indicating that the lambs had drowned. Additionally, there was one case of trauma, which involved a large lesion on the head and a haematoma on the right side of the thoracic cage. The sixth case was due to hypothermic starvation syndrome, characterised by a lack of colostrum intake and the presence of meconium in the intestines. The remaining 16 carcases showed severe lesions on numerous organs, suggesting an infectious cause of death (Table 4) and the presence of a small quantity of pus on three umbilici. The anatomopathological and histopathological studies of these organs demonstrated an acute inflammatory reaction with the primary markers of the vasculo‐exudative and cellular phase (active congestion, oedema and diapedesis of inflammatory cells with different stages of evolution). The most developed lesions were observed on the lungs, liver, kidney and heart, followed by the spleen, muscles and intestines (Figure 1), suggesting that this mortality was due to respiratory infections and omphalitis, complicated by septicaemia, and that the pathogen's main entry sites were the respiratory tract and umbilicus.

**TABLE 4:** Anatomopathological and histopathological findings in the affected organs.

Organs	Lesion assessment		Diagnosis
	Macroscopic description	Microscopic description	
Lungs (*n* = 15)	Congestion, haemorrhage, oedema, presence of abscesses, fibrinous deposit and hepatisation	Congestion, infiltration of the tissues with erythrocytes, inflammatory cells such as neutrophils, and exudation. The loss of the structural components of the normal tissue and diffuse cell destruction (degeneration and necrosis) associated with fibrinous deposits that replaced functional tissues. In the abscess location, we have cellular debris, inflammatory cells, and what appeared to be clusters of microorganisms	Acute pneumonia
Liver (*n* = 14)	Congestion, presence of abscesses, hard consistency upon palpation, and dark or pale colouration		Acute hepatitis
Kidney (*n* = 14)	Congestion, haemorrhage on the fascia, pale colouration on the cortex, and the presence of multiple cysts		Glomerulonephritis Epithelial nephritis
Heart (*n* = 13)	Congestion, discolouration, haemorrhage on the epicardial fat and the heart base, thickening of the pericardium layer, presence of small points, and necrosis on the atriums and the auricles		Acute myocarditis
Spleen (*n* = 4)	Congestion, pale colouration and muddy consistency		Acute splenitis
Muscle (*n* = 3)	Congestion and haemorrhage		Acute myositis
Abomasum (*n* = 1)	Congestion and haemorrhage, and the presence of black content		Abomasitis
Intestines (*n* = 1)	Congestion and haemorrhage		Acute enteritis

### Microbiological study

#### Bacterial isolation and identification

A total of 218 bacterial strains were isolated from the affected organs of the 16 carcases. The identification of these strains was confirmed using API 20E and MALDI‐TOF‐Microflex mass spectrometry, which gave excellent scores, ranging from 2.00 to 3.00. The pathogens involved in this mortality belonged to two families: Enterobacteriaceae (*n* = 189) and non‐fermenting Gram‐negative bacilli (*n* = 29) (Table 5).

**TABLE 5:** Distribution of isolated strains in organs.

		Isolated strains	
Organs	Number	Enterobacteriaceae	Non‐fermenting Gram‐negative bacilli
Lungs	15	*E. coli, K. pneumoniae, K. oxytoca, P. mirabilis, P. stuartii, E. cloacae, C. freundii*	*P. aeruginosa*
Liver	14	*E. coli, K. pneumoniae, K. aerogenes*, *K. oxytoca, P. mirabilis, P. stuartii*	*P. aeruginosa, A. pittii*
Kidney	14	*E. coli, E. fergusonii, K. pneumoniae, P. mirabilis, E. cloacae*	*P. aeruginosa, Achromobacter timonensis, A. variabilis, A. baumannii*
Heart	13	*E. coli, E. fergusonii, K. pneumoniae, P. mirabilis, P. stuartii, E. cloacae*	*P. aeruginosa, Comamonas kerstersii*
Trachea and tracheal swab	23	*E. coli, K. pneumoniae*, *P. mirabilis, P. stuartii, E. cloacae, Enterobacter spp., E. kobei, C. sedlakii*	*P. aeruginosa, A. lactucae*
Spleen	4	*E. coli, K. pneumoniae, K. aerogenes*	**/**
Muscles	3	*E. coli, E. cloacae*	**/**
Peritoneum swab	2	*E. coli, K. pneumoniae, K. aerogenes*	*P. aeruginosa*
Intestines	1	*E. coli*	**/**
Abomasum content	1	*E. coli*	**/**
**Total**	**90**	**189**	**29**

For the first time, we isolated *P. stuartii* in the case of a lamb with immediate post‐natal mortality, besides other opportunist pathogens of different genera (*Citrobacter, Comamonas* and *Acinetobacter*). The distribution of the main isolated strains in organs showed that the most infected organs were the lungs and trachea (respiratory tract) (Enterobacteriaceae [*n* = 62/189], non‐fermenting Gram‐negative bacilli [*n* = 11/29]); liver (Enterobacteriaceae [*n* = 42/189], non‐fermenting Gram‐negative bacilli [*n* = 5/29]), as well as the kidney (Enterobacteriaceae [*n* = 34/189], and non‐fermenting Gram‐negative bacilli [*n* = 7/29]) (Tables 6 and 7).

**TABLE 6:** Number of isolated strains (Enterobacteriaceae).

	Number of isolated bacterial strains (Enterobacteriaceae)											
Organs	*E. coli*	*E. fergusonii*	*K. pneumoniae*	*K. aerogenes*	*K. oxytoca*	*Proteus mirabilis*	*Providencia stuartii*	*Enterobacter cloacae*	*Enterobacter* spp.	*Enterobacter kobei*	*Citrobacter freundii*	*Citrobacter sedlakii*
Lungs	15	/	7	/	2	2	5	1	/	/	1	/
Trachea and tracheal swab	5	/	6	/	/	4	7	2	3	1	/	1
Liver	18	/	14	2	1	6	1	/	/	/	/	/
Kidneys	19	1	10	/	/	3	/	1	/	/	/	/
Heart	17	1	5	/	/	2	2	1	/	/	/	/
Spleen	3	/	3	1	/	/	/	/	/	/	/	/
Muscles	4	/	/	/	/	/	/	2	/	/	/	/
Peritoneum swab	3	/	2	2	/	/	/	/	/	/	/	/
Intestines	2	/	/	/	/	/	/	/	/	/	/	/
Abomasum content	1	/	/	/	/	/	/	/	/	/	/	/
Total strains	87	2	47	5	3	17	15	7	3	1	1	1
**Total**	**189**											

**TABLE 7:** Number of isolated strains (non‐fermenting Gram‐negative bacilli).

	Number of isolated bacterial strains (non‐fermenting Gram‐negative bacilli)						
Organs	*Pseudomonas aeruginosa*	*Comamonas kerstersii*	*Achromobacter timonensis*	*Acinetobacter variabilis*	*Acinetobacter lactucae*	*Acinetobacter pittii*	*Acinetobacter baumannii*
Lungs	3	/	/	/	/	/	/
Trachea and tracheal swab	7	/	/	/	1	/	/
Liver	4	/	/	/	/	1	/
Kidney	4	/	1	1	/	/	1
Heart	4	1	/	/	/	/	/
Spleen	/	/	/	/	/	/	/
Muscles	/	/	/	/	/	/	/
Peritoneum swab	1	/	/	/	/	/	/
Intestines	/	/	/	/	/	/	/
Abomasum content	/	/	/	/	/	/	/
Total strains	23	1	1	1	1	1	1
**Total**					**29**		

#### Antimicrobial resistance tests

From 218 isolates, we noted different resistance profiles, summarised as follows: Enterobacteriaceae (*n* = 189/218) and *E. coli* strains showed high rates of resistance to tetracycline (86.2%), amoxicillin (82.8%), amoxicillin/clavulanic acid (77%), trimethoprim/sulfamethoxazole (68.9%), and *E. fergusonii* to tetracycline (100%) and amoxicillin (100%). A high frequency of resistance among *K. pneumoniae* isolates was noted to amoxicillin (97.9%), amoxicillin/clavulanic acid (85.1%), fosfomycin (74.5%), trimethoprim/sulfamethoxazole (72.3%), piperacillin/tazobactam (55.3%) and tetracycline (53.2%). *K. aerogenes* were found to be resistant to amoxicillin (100%), amoxicillin/clavulanic acid (80%), trimethoprim/sulfamethoxazole (80%) and tetracycline (60%), and *K. oxytoca* to amoxicillin (100%) and amoxicillin/clavulanic acid (66.7%). Furthermore, resistance of *P. mirabilis* was noted to tetracycline (88.2%) and *P. stuartii* to amoxicillin (93.3%), trimethoprim/sulfamethoxazole (93.3%), tetracycline (93.3%), amoxicillin/clavulanic acid (80%) and fosfomycin (66.7%). All strains of *Enterobacter* spp., *E. kobei, C. sedlakii*, *E. cloacae* and *C. freundii* were resistant to amoxicillin (100%) and amoxicillin/clavulanic acid (100%), in addition to tetracycline resistance for *E. cloacae* (57.1%) and total resistance to both tetracycline and ciprofloxacin for *C. freundii* (Table 8).

**TABLE 8:** Antibiotic resistance rates (%) of the isolated strains (Enterobacteriaceae).

Isolates	Percentage of the Enterobacteriaceae strains showing resistance (%)															
	AMX20	AMC30	FEP30	TPZ36	MEC10	CRO30	ETP10	IPM10	FF200	FT100	SXT25	AK30	CIP5	TET30	CS50	GN10
*E. coli*	82.8	77	2.3	16.1	8	2.3	/	/	3.4	2.3	68.9	3.4	11.5	86.2	2.3	4.6
*E. fergusonii*	100	/	/	/	/	/	/	/	/	/	/	/	/	100	/	/
*K. pneumoniae*	97.9	85.1	21.3	55.3	44.7	19.1	/	/	74.5	2.1	72.3	2.1	6.4	53.2	4.3	4.3
*K. aerogenes*	100	80	20	/	40	20	/	/	40	/	80	/	/	60	/	/
*K. oxytoca*	100	66.7	/	/	33. 3	/	/	/	/	/	33.3	/	/	/	/	/
*Proteus mirabilis*	11.8	5.9	5.9	/	/	/	/	/	11.8	100	5.9	/	/	88.2	100	5.9
*Providencia stuartii*	93.3	80	/	/	13.6	/	/	/	66.7	93.3	93.3	13.6	/	93.3	100	/
*Enterobacter cloacae*	100	100	14.3	/	/	/	/	/	42.9	28.6	28.6	/	/	57.1	/	/
*Enterobacter* spp.	100	100	/	/	/	33. 3	/	/	/	33.3	33.3	/	/	/	/	/
*Enterobacter kobei*	100	100	/	/	/	/	/	/	/	/	/	/	/	/	/	/
*Citrobacter freundii*	100	100	/	/	/	/	/	/	/	/	/	/	100	100	/	/
*Citrobacter sedlakii*	100	100	/	/	/	/	/	/	/	/	/	/	/	/	/	/

For non‐fermenting Gram‐negative bacilli (*n* = 29/218), *P. aeruginosa* showed a high resistance rate to minocycline (100%), trimethoprim/sulfamethoxazole (91.3%), and rifampicin (78.3%). All (100%) of the *Comamonas kerstersii* isolates showed full resistance to aztreonam, ceftazidime, fosfomycin, rifampicin, trimethoprim/sulfamethoxazole, amikacin and ciprofloxacin. All *A. variabilis* isolates were resistant to trimethoprim/sulfamethoxazole. All (100%) of the *A. pittii* isolates were resistant to piperacillin/tazobactam, aztreonam and fosfomycin, and all *A. baumannii* isolates were resistant to aztreonam (Table 9). *A. lactucae* and *Achromobacter timonensis* showed no resistance to any of the tested antibiotics (Table 9).

**TABLE 9:** Antibiotic resistance rates (%) of the isolated strains (non‐fermenting Gram‐negative bacilli).

Isolates	Percentage of non‐fermenting Gram‐negative bacilli strains showing resistance (%)															
	TIC 75	TIM 85	TPZ 36	ATM 30	CAZ 10	FEP 30	MER 10	IPM 10	FF 200	RA 5	SXT 25	AK 30	CIP 5	MIN 30	CS 50	GN 10
*Pseudomonas aeruginosa*	4.3	8.7	4.3	/	/	/	/	/	/	78.3	91.3	/	/	100	4.3	4.3
*Comamonas kerstersii*	/	/	/	100	100	/	/	/	100	100	100	100	100	/	/	/
*Achromobacter timonensis*	/	/	/	/	/	/	/	/	/	/	/	/	/	/	/	/
*Acinetobacter variabilis*	/	/	/	/	/	/	/	/	/	/	100	/	/	/	/	/
*Acinetobacter lactucae*	/	/	/	/	/	/	/	/	/	/	/	/	/	/	/	/
*Acinetobacter pittii*	/	/	100	100	/	/	/	/	100	/	/	/	/	/	/	/
*Acinetobacter baumannii*	/	/	/	100	/	/	/	/	/	/	/	/	/	/	/	/

#### Real‐Time PCR

The characteristics of the strains isolated from the lamb carcases showed the presence of only the ESBL gene *bla*_SHV_ in *Klebsiella* spp. strains. That confers resistance to many beta‐lactam antibiotics, such as penicillins and cephalosporins.

## DISCUSSION

This study involved 35 sheep farms located in the province of Constantine, northeastern Algeria. The selection of the farms was based on the owners’ willingness to cooperate with the study and to spend a significant amount of time and effort to collect the various lamb carcases. The objective was to provide epidemiological data on neonatal lamb mortality, determine the main risk factors by statistical analysis, understand the implicated bacterial pathogens, and assess lesions. The registered mortality rate was 5.74%, higher than the rate from Norwegian surveys conducted between 2000 and 2010, which increased from 3.3% to 4.7%,^32^ and lower than the majority of recorded rates in Europe, such as France (18.4%)^12^ and England (9.7%)^33^; in America, such as Canada (15.8%)^34^ and the United States (15.5%)^35^; and in Algeria itself in Bechar (14.35%),^9^ Tebessa (25.09%)^13^ and Tiaret (24%).^11^ According to Fragkou et al., in a well‐managed sheep flock, the mortality rate should be around 3%, with a maximum limit of 5%.^36^

In the current study, the low rate recorded (slightly above the maximum limit of 5%) does not accurately represent the neonatal mortality rate in the study region. Due to economic constraints, we focused our sampling on municipalities with easily accessible farms and cooperative owners who agreed to participate voluntarily in the study. Thus, those who declined to participate may have experienced higher mortality, suggesting a lack of effective management practices.^37^

The statistical analysis of the collected data revealed that the hygiene of soil and livestock buildings is the principal risk factor involved in lamb deaths. This is explained by the fact that according to numerous studies carried out in Morocco,^38^ the UK,^39^ and the United States,^40^, ^41^ poor hygiene increases the probability of exposure to several pathogens and thus increases the risk of lamb mortality. Meanwhile, the breeding system (reproduction system, housing ewes according to their stage [empty/full], housing lambs according to their age) and the hygiene of the soil and the livestock buildings determine the type of mortality (infectious or non‐infectious). Indicating that preparing ewes for gestation and lambing includes adapting feed rations to their growing needs, appropriate monitoring practices, and full support for ewes and newborn lambs, is crucial to increase lambs' survival.^12^ Moreover, lamb mortality was recorded during the lambs’ first month of life, which corroborates the findings of Wilson et al., suggesting that during this period, the immune system of lambs is still developing, which makes newborns particularly vulnerable to infections.^42^, ^43^

The anatomopathological and histopathological findings show that the main injured organs were the lungs, liver, kidney and heart, followed by the spleen, muscles and digestive tract (abomasum and intestines). The majority of the infectious deaths were attributed to respiratory disorders and omphalitis complicated by septicaemia. Meanwhile, non‐infectious causes of death included stillbirth, traumatic lesions and hypothermia starvation syndrome.

The causes of neonatal mortality vary worldwide. In Australia and New Zealand, studies from 1970 to 2022 all concluded that the leading causes of lamb losses were non‐infectious, including dystocia, starvation, mismothering, stillbirth and trauma, followed by the involvement of infectious agents (*E. coli, Sphaerophorus necrophorus, Pasteurella haemolytica* and *Pasteurella multocida*).^44^, ^45^, ^46^, ^47^, ^48^ On the other hand, in Norway, India, Pakistan, Brazil and Ethiopia, infections were identified as the most significant cause of mortality (pneumonia, gastrointestinal affections and septicaemia), followed by non‐infectious causes.^49^, ^50^, ^51^, ^52^, ^53^, ^54^ In France, farmers reported both infectious and non‐infectious causes, including enterotoxaemia, diarrhoea, respiratory tract infections, omphalitis and septicaemia for the former, and dystocia, drowned lambs and weak lambs at birth for the latter.^6^, ^12^ In Algeria, studies have described only non‐infectious causes such as dystocia (abnormal presentations, cervical problems and uterine torsion) and neonatal diarrhoea as contributors to lamb mortality.^8^, ^55^

Histopathology revealed that the most developed lesions were found in the lung, indicating that the respiratory tract and umbilicus are the sites of primary infection in our infectious death cases, and that lesions in other organs are the result of a septicaemic evolution. In their studies in Egypt, Wassif and El‐Kattan revealed that the lungs and liver are the main organs affected in lamb deaths.^56^, ^57^ Another Indian study by Raghavendra et al. reported that the principal lesions were located in the lungs, liver, kidneys and intestines.^58^

Microbiology evaluation concluded that the pathogens implicated in this neonatal lamb mortality belonged to two different families: Enterobacteriaceae and non‐fermenting Gram‐negative bacilli. Various studies have shown that isolated strains can cause pneumonia and sepsis, especially in newborn people^59^ and animals.^56^, ^57^ In animal studies on the respiratory tract of sheep, *E. coli, K. pneumoniae*, *E. cloacae* and *P. aeruginosa* were frequently isolated in cases of pneumonia, with or without the formation of lung abscesses at different rates.^56^, ^57^, ^60^, ^61^, ^62^, ^63^ However, *E. cloacae* and *K. oxytoca* were implicated in the septicaemic evolution of infections.^60^, ^61^

In Egypt and Turkey, studies showed that *Klebsiella pneumoniae*, *Mannheimia haemolytica, E. coli* and *P. multocida* were involved in lamb pneumonia,^56^, ^57^, ^64^ and in India, *E. coli* caused septicaemic colibacillosis, corroborating our results.^65^ But no reports indicate that other species, such as *E. fergusonii*, *C. freundii*, *C. sedlakii* and *Acinetobacter* spp., were involved in lamb infections as in the current study. According to some research, they may be linked to septicaemia, respiratory illnesses, diarrhoea and encephalitis in pigs, horses,^66^ sheep,^67^ dogs^68^ and aquatic animals.^69^ Our study also reported the presence of *P. stuartii*. To our knowledge, clinical cases involving this pathogen are exceedingly uncommon, especially in lambs. However, its association with bronchopneumonia has been reported in sheep,^70^ sepsis in primates,^71^ and ulcerative dermatitis and cellulitis in dogs.^72^ Furthermore, *Providencia* spp. has been detected in meat sold in China and Japan.^73^ This could lead to serious public health problems, as this microorganism has been rarely described in the veterinary literature. Despite *C. kerstersii* being considered non‐pathogenic, recent reports have shown that in people, it can cause bacteraemia in young patients and complications such as abdominal pain, chills and rigours in older ones.^74^, ^75^, ^76^ In animals, aside from its isolation in our investigation, *C. kerstersii* was only involved in one case of urinary tract infection in a 7‐month‐old goat.^77^

Regarding antimicrobial susceptibility, most of the isolated strains exhibited antimicrobial resistance to at least two or three antibiotics. *E. coli* strains exhibited a high resistance rate to amoxicillin, amoxicillin/clavulanic acid, trimethoprim/sulfamethoxazole and tetracycline, and *K. pneumoniae* isolates to amoxicillin, amoxicillin/clavulanic acid, fosfomycin, trimethoprim/sulfamethoxazole, piperacillin/tazobactam and tetracycline, which are the most commonly used drugs to treat lamb infections on the studied farms, thus explaining the therapeutic failure and lamb mortality rate. According to Egyptian investigations on sheep pneumonia, *Klebsiella* spp. and *E. coli* isolates were mainly resistant to trimethoprim/sulfamethoxazole and tetracycline, which are the most commonly used drugs in this region to treat respiratory tract infections.^57^, ^61^ For non‐fermenting Gram‐negative bacilli, *P. aeruginosa* isolates were resistant to minocycline, trimethoprim/sulfamethoxazole and rifampicin. In an Iraqi study, *P. aeruginosa* strains were resistant to ampicillin, aztreonam, ceftazidime and levofloxacin.^60^

The present study also demonstrated that only the *Klebsiella* spp. isolates harboured the *bla*_SHV_ genes from lambs’ infected organs. In 2019, Ali and Abu‐Zaid reported the presence of *bla*_CTX‐M_ *, Sul1, bla*_TEM_ *, tetA* and *bla*_SHV_ genes in *K. pneumoniae* isolates from sheep and goats. In 2013, Li et al. also reported that *K. pneumoniae* infections are frequently multidrug resistant, involving strains that produce ESBL genes (*bla*_TEM_, *bla*_SHV_ and *bla*_CTX‐M_ types).^78^ The findings of this study demonstrate the rise of opportunistic pathogens and the ineffectiveness of broad‐spectrum antibiotics such as tetracycline, trimethoprim/sulfamethoxazole, amoxicillin and amoxicillin/clavulanic acid in treating neonatal infections.

Despite the small sample size of carcases, the low participation rate, and the use of convenience sampling, this study is a first step in gathering initial data on the epidemiological status of the sheep flock in the Constantine region. Further investigations into the pathogens associated with lamb mortality are crucial, given the rise of several opportunistic pathogens with alarming resistance rates. This pathogenicity requires clarification through research on virulence genes, which could elucidate the role of each pathogen in the infectious processes leading to this mortality. Furthermore, the creation of farm‐specific epidemiological models will enable the implementation of effective management strategies. This includes appropriate treatment, rapid diagnosis and prophylaxis, which will ultimately enhance the overall health of the flock both in the region and globally.

## CONCLUSION

This study demonstrated that the mortality rate is slightly above the acceptable limit, associated with the hygiene of the soil, livestock buildings, and the breeding system, which are the main risk factors in neonatal lamb mortality in Constantine (Algeria). Postmortem examination and histopathological findings revealed that respiratory disorders are the principal cause of death, followed by omphalitis, both complicated by septicaemia, in addition to stillbirths, traumatic lesions and hypothermia starvation syndrome. Isolated strains belonged to the Enterobacteriaceae and the non‐fermenting Gram‐negative bacilli groups. The main isolated species were *E. coli, K. pneumoniae* and *P. aeruginosa*, frequently isolated in sheep pneumonia. However, the results also revealed the involvement of opportunistic pathogen genera, such as *Providencia*, *Citrobacter*, *Comamonas* and *Acinetobacter*, in lamb mortality, which should encourage further research on virulence genes to clarify their role in the infectious processes that lead to death. Antimicrobial resistance tests confirmed that the majority of the isolates are resistant to broad‐spectrum antibiotics (amoxicillin, amoxicillin/clavulanic acid, trimethoprim/sulfamethoxazole and tetracycline), which may explain the therapeutic failure as well as the septicaemic evolution of cases. Based on these findings, we recommend that the investigated farms implement preventive sanitary practices (cleaning the mothering and lambing pens, changing their litter, and cleaning all materials that may come into contact with the newborns) to reduce exposure to opportunistic pathogens. We also suggest assisted lambing, proper management of lambs during their first days of life, prompt diagnosis, and antimicrobial sensitivity testing, which will enable appropriate treatment and help prevent fatal outcomes of infections in lambs. Furthermore, to effectively control these mortality rates and provide more valuable data on newborn lamb mortality, developing farm‐specific epidemiological modelling is required, as well as investing in training programmes to help farmers put more efficient monitoring practices in place.

## AUTHOR CONTRIBUTIONS

All listed authors’ contributions include the conception and design, acquisition of data or analysis and interpretation of data, drafting the article or revising it critically for important intellectual content, and final approval of the published version. Regarding responsibility for overall content, the lead author, Benmebarek Hayem, is the guarantor.

## CONFLICTS OF INTEREST

The authors declare they have no conflicts of interest.

## ETHICS STATEMENT

This study was approved by the El Khroub Scientific Council of the Institute of Veterinary Sciences of Constantine 1 Frères Mentouri University. Authorisations for the collection of lamb carcases (natural deaths) and the transfer of samples (bacterial strains and histological slides) to the Institut Hospitalo‐Universitaire (IHU) of Marseille, France, were issued under reference numbers 145/22 and 1072/22, respectively.

## Data Availability

The data that support the findings of this study are available from the corresponding author upon reasonable request. Unpublished data are not available.
